# Effects of the urban heat island on the phenology of Odonata in London, UK

**DOI:** 10.1007/s00484-017-1311-7

**Published:** 2017-02-11

**Authors:** Giovanna Villalobos-Jiménez, Christopher Hassall

**Affiliations:** School of Biology, University of Leeds, Woodhouse Lane, Leeds, LS2 9JT UK

**Keywords:** Climate change, Dragonflies, Damselflies, Life history, Flight period, Aquatic ecosystems

## Abstract

Urbanisation is one of the major drivers of ecosystem change and includes increased temperatures in cities leading to an urban heat island (UHI). This study quantified the phenological response of odonates across London, UK, from 1990 to 2012, using a database of 1,031,277 historical sightings. The ordinal flight dates of each species were used to calculate the leading edge, middle and trailing edge of the flight period (P5, P50 and P95, respectively). The results suggest that the phenology of odonates is affected by the UHI only at a community level: no significant changes in the P5 or P50 of the flight period were found, although the P95 shows a mean advance of 4.13 days compared to rural areas, thus suggesting a contraction of the flight period in urban areas. However, only one individual species (*Sympetrum striolatum*) exhibited an advance in the P95 of the flight period in urban areas compared to rural areas. On the other hand, climate change (minimum temperature) had a much stronger impact on the phenology of odonates at the community level with a significant advance of 6.9 days °C^−1^ in the P5 of the flight period, 3.1 days °C^−1^ in the P50 and 3.3 days °C^−1^ in the P95 flight date. Similarly, a significant advance in P5 was found in 7 of the 15 species tested in response to minimum temperature, and 2 species showed a significant advance in P50 in response to minimum temperature, but no species showed a shift in the P95 flight date due to minimum temperature. As shown in previous studies, life history influences the phenological response of odonates, with spring species and those species lacking an egg diapause being the most responsive to increased temperatures, although summer species and species with obligate egg diapause also respond to the UHI by advancing the P95 by 3.8 and 4.5 days, respectively, compared to rural areas, thus contracting the flight period. The present study shows that the UHI has negligible impacts on emergence patterns of odonates compared to climate change, which may result from the capacity of aquatic habitats to buffer the microclimatic conditions of the surrounding terrestrial habitats. We conclude by highlighting the importance of climate change on freshwater habitats over the impacts of the UHI.

**Electronic supplementary material:**

The online version of this article (doi:10.1007/s00484-017-1311-7) contains supplementary material, which is available to authorized users.

## Introduction

With over 50% of people now living in cities (Grimm et al. 2008) and the impacts extending beyond the metropolitan borders (Faeth et al. 2011), studying the effects of urbanisation has become crucial in order to understand urban ecosystems and mitigate the negative impacts of cities. The many impacts of urbanisation include a significant increase in temperature in urbanised areas compared to the rural surroundings, referred to as the “urban heat island” (UHI) effect. This effect is mainly caused by the increased areas of dark, impervious surfaces in cities, as well as the low abundance of vegetation (Jochner and Menzel 2015). The increase in temperature ranges on average from 0.5 to 3.0 °C depending on weather conditions (Jochner and Menzel 2015), although the strength of the UHI effect is also positively correlated with city size (Oke 1973). The effect of the UHI is greater during the night (Karl et al. 1988) and even more so in winter (Parker 2004). The UHI effect also increases water temperature by transferring heat from rooftops and asphalt roads to storm water runoff, leading to increased thermal pollution once it enters water bodies (Jones et al. 2012).

Such a substantial change in local climate is likely to have knock-on effects for biological processes. One of the most common indicators of climate variation is the timing of biological events, known as “phenology” (Menzel et al. 2006; Walther et al. 2002), and this phenomenon has been acknowledged as an indicator of changing climate by the UK Government (Cannell et al. 1999). Over 80% of terrestrial, marine and freshwater species in UK have advanced their phenology due to climate change (Thackeray et al. 2010), while at a global scale, almost 60% of the species studied (including plants, birds, butterflies and amphibians) showed significant changes in their phenology over the past 30 to 150 years (Parmesan and Yohe 2003), which suggest that phenological responses to climate change are highly coherent at a global scale. Likewise, phenology is also influenced by the UHI effect. It has been shown that the UHI advances the flowering and leaf unfolding of plants (e.g. Jochner et al. 2013) and the reproductive phenology of birds (Deviche and Davies 2014) and amphibians (Cook et al. 2006). However, currently, there is a lack of studies regarding the impact of the UHI effect on aquatic organisms.

Dragonflies and damselflies (Odonata) represent an aquatic insect group which has been proposed as a candidate barometer of climate change (Hassall 2015). British odonate species have advanced their flight season by 1.5 days each decade (Hassall et al. 2007). In the Netherlands, odonates have also advanced their flight seasons towards the spring (Dingemanse and Kalkman 2008). The phenological response of odonates to increasing temperatures has also been studied in the laboratory: larvae reared at 5 °C above ambient temperature had emerged approximately 3 weeks before the larvae reared at ambient temperature (McCauley et al. 2015). Predictive models also suggest that the emergence of *Gomphus vulgatissimus* is likely to advance by 6–7 days per 1 °C increase (Richter et al. 2008), especially at higher latitudes where increased development speed is expected to occur due to climate change (Braune et al. 2008). The impact of climate change on the phenology of odonates is explained by the fact that their life history is greatly influenced by temperature (Hassall and Thompson 2008). First, temperature dictates the success and duration of the embryonic development (Pilon and Masseau 1984) and egg diapause, a period of developmental stasis (Sawchyn and Church 1973). Second, increased temperatures tend to accelerate the growth rate of larvae (Pritchard et al. 2000) and also play a key role in ecdysis (Lutz 1974). In the adult phase, warm temperatures also increase reproductive success (Banks and Thompson 1987).

This study examines the phenology of odonates in the context of the UHI in the city of London, UK. We hypothesise that, as is the case with temporal trends in climate, higher temperatures caused by the UHI will lead to an advance in the phenology of odonates in urban areas relative to the surrounding non-urban areas. This phenological advance would be reflected in earlier observations of adult odonates in the city compared to rural areas.

## Methods

**Fig. 1:**
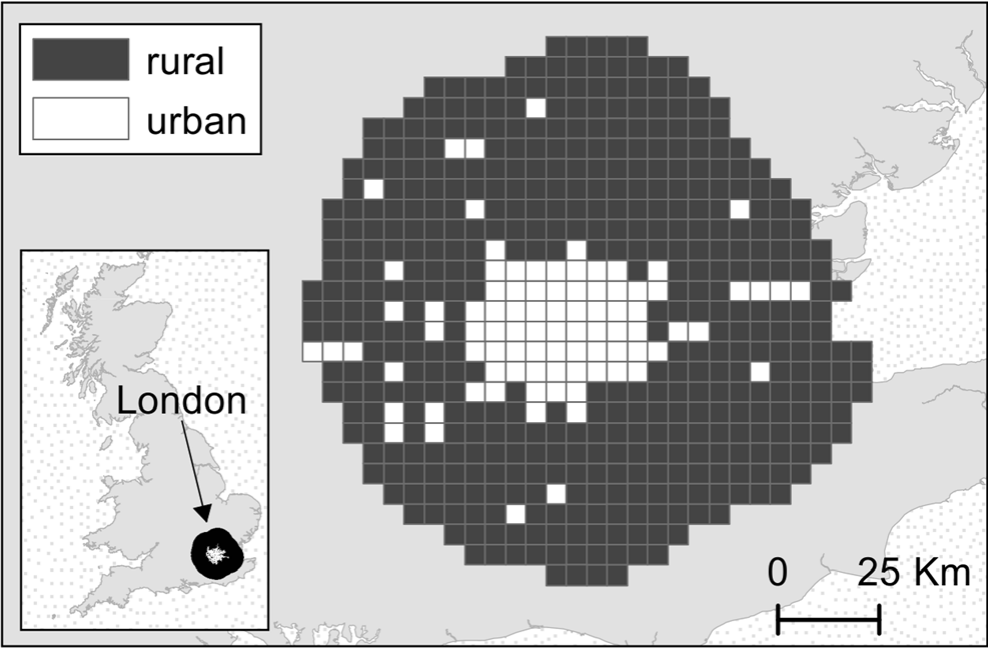
Location of urban (*white*) and rural (*black*) grid squares sampled for the phenological analysis in London, UK

**Fig. 2:**
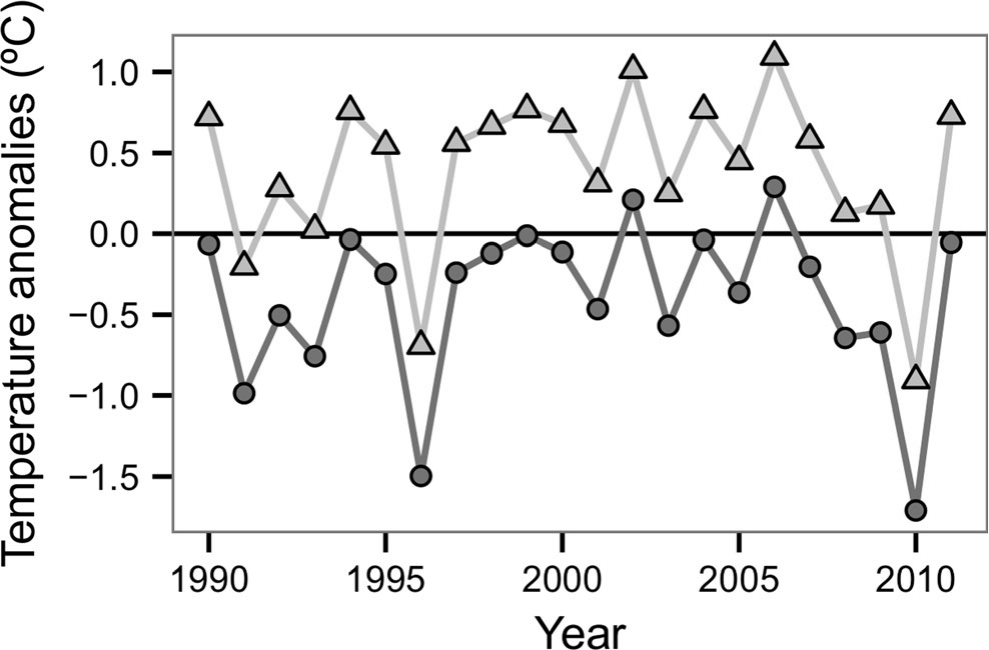
Anomalies in minimum temperature in urban (*triangle*) and rural (*circle*) areas sampled from 1990 to 2012

The urban area of London and the surrounding rural areas were extracted from the Ordnance Survey (OS) Meridian 2 data (Ordnance Survey 2013). London was chosen as a study site for a number of reasons: (i) the size of the city means that it should have a high degree of urban warming; (ii) recording of odonates is greatest in the south of the UK and so there should be a large number of records; and (iii) there is a moderately rich odonate fauna present in the south of England which allows straightforward identification. The “developed land use area” (DLUA) layer was used from the OS Meridian 2 data to define the extent of the urban area. A buffer of 40 km was created around the city polygon, and the sample area was defined as a 5 km × 5 km grid (540 grid cells in total), since climate data was also available at this resolution. Grid squares were defined as urban depending on whether over 50% of the grid cover was within the DLUA region (Fig. 1). Once the urban and rural areas were defined, the annual mean minimum temperatures for each grid square were calculated from the UKCP09 gridded data sets (UK Climate Projections 2009; Fig. 2). Air temperatures not only are influential during the adult phase of odonates but also tend to be correlated to surface water temperatures (Livingstone and Lotter 1998; McCombie 1959), which influence the development of the larvae, and therefore are suitable measures for predicting the phenology of odonates. Moreover, air temperatures have already been used as a surrogate for studying phenological shifts in odonates (e.g. Hassall et al. 2007). In this study, minimum temperature was selected as a quantitative measurement of the UHI effect because (i) the increase in temperature in cities is greater at night when the temperatures are cooler (Karl et al. 1988); (ii) atmospheric and surface temperatures in cities show similar patterns during the night (U.S. Environmental Protection Agency 2008); (iii) night temperatures are not influenced by solar radiation; and (iv) the development of insects is more likely to be hindered by lower temperatures which may fall below critical thermal thresholds. The urban grids sampled were significantly warmer than the rural grids by 0.8 °C (*t* = −47.01, df = 3733.764, *P* < 0.001). From this step onwards, the analysis was performed in R 3.0.2 (R Core Team 2013).

To analyse phenological shifts, the British Dragonfly Society (BDS) database was used, which contains 1,031,277 records of sightings of odonates (date accessed: 20/01/2015). Species considered migratory or not established in the UK according to the BDS species list (http://www.british-dragonflies.org.uk) were excluded a priori. Furthermore, only adult records within the urban and rural grid squares with an exact flight date from 1990 to 2012 were selected. This period of time was chosen to minimise temporal variation in urban development and in temperature.

Ordinal dates from records for each species were collated for each year from rural and urban sites. Three percentiles (P5, P50 and P95, corresponding to the leading edge, middle and trailing edge of the flight period, respectively) were calculated from the distributions of these ordinal dates. However, only species with over 30 records in each type of land use per year in a period of at least 10 years were used to ensure the percentiles were representative of the true flight-period trends. This led to the selection of 96,366 records from the following species: *Aeshna cyanea*, *Aeshna grandis*, *Aeshna mixta*, *Anax imperator*, *Calopteryx splendens*, *Coenagrion puella*, *Enallagma cyathigerum*, *Erythromma najas*, *Ischnura elegans*, *Libellula quadrimaculata*, *Orthetrum cancellatum*, *Pyrrhosoma nymphula* and *Sympetrum striolatum*.

The BDS database represents a valuable collection of observations of odonates from volunteers throughout UK. However, recording is executed without a standardised sampling method, resulting in uneven recorder effort, which may bring some complications for any analysis (Hassall and Thompson 2010). In the present study, the data showed more records from rural areas. Given that the amount of records was highly unbalanced between urban and rural areas and the data was heteroskedastic, a feasible generalised least squares (FGLS) model was used (Gregorie 1987) to test whether the flight season of each species was more advanced in the city compared to rural areas. Each of the percentiles by species was used as the response variable, whereas land use (urban vs rural), year and minimum temperature were used as the predictor variables. However, minimum temperature and year are expected to be collinear variables as a result of climate change. Therefore, to test for multicollinearity, variance inflation factors (VIFs) were used. All *P* values were adjusted using the false discovery rate (FDR) correction to account for the number of tests executed (1 test for each of the 3 percentiles across 13 species gives a total of 39 tests).

To assess the impact of the UHI on community-level phenology shifts (i.e. pooling all Odonata), the coefficients from the FGLS models (year, temperature and urban land use) were tested using a one-sample, two-tailed *t* test (Hassall et al. 2007). A significant difference from zero in the mean change in flight dates would imply a phenological shift across all the species tested. All nine *P* values obtained from the community-level tests were also adjusted using the FDR correction. In order to take into account the inherent correlation of traits among closely related species, a phylogenetic approach is necessary. However, phylogenetic comparative methods require a reliable phylogeny (Felsenstein 1985; O'Meara 2012) and, despite the great efforts to describe the phylogeny of European odonates, such phylogeny has not been fully resolved (Dijkstra and Kalkman 2012). In such conditions, ideally the evolutionary correlation of traits would be partially controlled using an ANOVA with type I (sequential) sum of squares (Hof et al. 2006). However, the sub-branching of life history traits in the phylogenetic groups considered in this study lead to such a high collinearity that the effects of phylogeny and life history were indiscernible between each other. Therefore, this study is focused exclusively on life history traits and we cannot rule out an effect of phylogenetic relatedness per se. Similar to the community-level phenological analysis in the present study, the coefficients from the FGLS were tested against their life history traits (Hassall et al. 2007) using a one sample, two-tailed *t* test and also adjusting the *P* values using the FDR correction to account for multiple testing. Such traits include the presence/absence of egg diapause (although facultative egg diapause was excluded considering it was represented by only one species, *S. striolatum*), as well as the classification of spring/summer species. The term “spring species”, coined by Corbet (1954), refers to odonates which have a larval diapause in the last instar of their life history and emerge during the spring in a synchronous manner, whereas “summer species” emerge asynchronously during the summer and, if a larval diapause is present, occur during any other instar. These life history traits were chosen since they influence the phenology directly and are likely to respond to the consequent temperature changes of the UHI.

Using adult records instead of emergence patterns offers various advantages for studying phenology, most importantly, the ability to evaluate changes throughout the flight period rather than only the leading edge of the flight period. However, adult odonates tend to disperse, and therefore, it is likely that an individual recorded in a site may not have developed and emerged there. Nevertheless, most species do not disperse more than 1 km from their emergence site (Angelibert and Giani 2003; Bennett and Mill 1995; Conrad et al. 1999; Stettmer 1996; Ward and Mill 2007) and though anisopterans are commonly known to have long-distance dispersal, particularly in the case of aeshnids, unfortunately, there is insufficient data to estimate the maximum distance dispersal in this group. Therefore, the phenology analysis at a community level was repeated but excluding species that are more likely to disperse long distances—the Anisoptera—to test the validity of the study. Accounting for life history, in this case, was not possible due to the fact that all zygopterans in this study have no diapause, and only three spring species and three summer species were left to compare these traits.

## Results

**Fig. 3:**
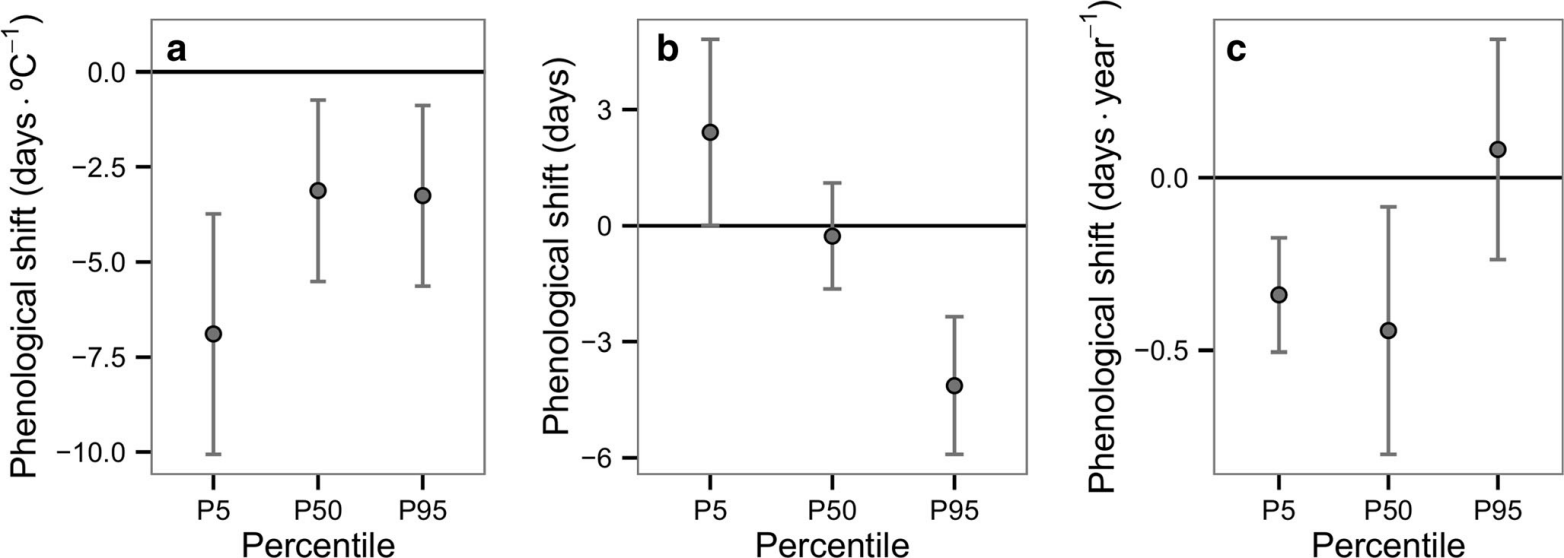
Community-level shifts in flight dates (*P5*, *P50*, *P95*) in relation to **a** minimum temperature, **b** urban land use compared to the rural surroundings and **c** year. *Error bars* represent 95% confidence intervals

Regarding the community-level shifts in response to urbanisation, a small but statistically significant advance of the P95 flight date of 4.1 days (*t* = −5.066, df = 12, *P* = 0.002) was found after using the FDR correction, and no significant change was found in P5 or P50 (P5: mean = 2.4 days, *t* = 2.190, df = 12, *P* = 0.063; P50: mean = −0.3 days, *t* = −0.429, df = 12, *P* = 0.675), representing a contraction of the flight period of odonates in cities (Fig. 3). However, in response to changing minimum temperature, a mean advance was observed in all flight dates at a community level, even after the FDR correction (P5: mean = −6.9 days °C^−1^, *t* = −4.748, df = 12, *P =* 0.002; P50: mean = −3.1 days °C^−1^, *t* = −2.855, df = 12, *P* = 0.026; P95: mean = −3.3 days °C^−1^, *t* = −2.988, df = 12, *P* = 0.025; Fig. 3). Regarding the phenological shifts by year, changes in the P5 and P50 flight dates at a community level were significant after using the FDR correction (P5: mean = −0.3 days year^−1^, *t* = −4.461, df = 12, *P* = 0.002; P50: mean = −0.4 days year^−1^, *t* = −2.694, df = 12, *P* = 0.029; P95: mean = 0.1 days year^−1^, *t* = 0.559, df = 12, *P* = 0.660). These results suggest that climate change is advancing the flight period, while the UHI is contracting the flight period.

After excluding the Anisoptera, the results confirm the community-level advance of the P5 and P95 flight dates in response to minimum temperature (P5: mean = −10.2 days °C^−1^, *t* = −13.087, df = 5, *P* < 0.001; P50: mean = −4.0 days °C^−1^, *t* = −2.275, df = 5, *P* = 0.094; P95: mean = −4.8 days °C^−1^, *t* = −7.736, df = 5, *P* = 0.003; see Fig. S1 in the Supplementary Material). In response to the UHI, the advance in the P95 flight date was confirmed (P5: mean = −0.1 days, *t* = −0.056, df = 5, *P* = 0.957; P50: mean = −1.7 days, *t* = −3.132, df = 5, *P* = 0.047; P95: mean = −4.1 days, *t* = −3.186, df = 5, *P* = 0.047; Fig. S1 in the Supplementary Material), which suggests a contraction of the flight period in the city. Regarding phenological shifts by year, only the advance in P50 flight date was confirmed (P5: mean = −0.3 days year^−1^, *t* = −2.265, df = 5, *P* = 0.094; P50: mean = −0.7 days⋅year^−1^, *t* = −4.749, df = 5, *P* = 0.015; P95: mean = 0.03 days year^−1^, *t* = 1.303, df = 5, *P* = 0.280; Fig. S1 in the Supplementary Material). It is worth mentioning, however, that even though all the *P* values were adjusted using the FDR correction, only six species were included for this part of the analysis, thus limiting statistical power. Nevertheless, these results suggest that the advance of the flight period of odonates in response to minimum temperature and the contraction of the flight period in response to the UHI are robust whether or not species with higher dispersal were included.

After the FDR correction, no significant effects of the UHI were found on the P5 or P50 flight dates of any individual species of odonates. However, there was a significant effect of urban land use found on the P95 flight date of only one species (*S. striolatum*), with an advance of 10.03 days in the city compared to surrounding rural areas (Table 1), thus shortening the flight period of this species in cities. On the other hand, minimum temperature had a significant effect in the flight dates of 7 of the 13 species tested (see Table 1). All the species affected by minimum temperature showed an advance in the P5 flight date and two of these species also showed an advance in the P50 flight date (Table 1). No species presented any significant difference in the P95 flight date due to minimum temperature. Phenological shifts across the years were shown to be statistically significant in eight species, of which two species showed an advance in the P5 flight date, four species advanced the P50 flight date, one species showed a delay in P50 (*S. striolatum*) and two species showed a delay in the P95 flight date (see Table 1 for details). The rates of the statistically significant shifts per year showed a maximum value of 0.97 days year^−1^ (Table 1). All models showed a low VIF value (see Table S1 in the Supplementary Material); therefore, the results of the models were not affected by multicollinearity.

**Table 1:** Estimates of the coefficients of year, minimum temperature and urban land use on the flight dates of each species analysed

		P5		P50		P95	
Species	Predictor	Coefficient	*P*	Coefficient	*P*	Coefficient	*P*
*Aeshna cyanea*	Year	−0.371	0.687	0.522	0.246	0.810	0.314
	Min. temp.	0.816	0.962	−3.457	0.687	0.895	0.962
	Land use	8.484	0.435	0.702	0.957	−4.629	0.701
*Aeshna grandis*	Year	−0.476	0.130	−0.186	0.378	0.113	0.701
	Min. temp.	−1.701	0.715	−3.955	0.131	−2.620	0.516
	Land use	5.595	0.198	4.000	0.142	−3.933	0.272
*Aeshna mixta*	Year	−0.581	0.077	0.307	0.169	*0.758*	*0.035*
	Min. temp.	2.567	0.602	−1.899	0.542	−0.959	0.886
	Land use	0.423	0.962	0.288	0.962	−4.956	0.313
*Anax imperator*	Year	−0.431	0.173	*−0.764*	*0.025*	−0.291	0.479
	Min. temp.	−5.966	0.108	1.830	0.701	2.877	0.583
	Land use	2.966	0.542	0.136	0.978	−1.766	0.745
*Calopteryx splendens*	Year	*−0.637*	*0.043*	−0.868	0.080	0.001	0.996
	Min. temp.	*−8.014*	*0.040*	1.030	0.923	−2.792	0.674
	Land use	2.381	0.636	−1.288	0.886	0.140	0.978
*Coenagrion puella*	Year	−0.011	0.968	−0.129	0.701	−0.047	0.957
	Min. temp.	*−11.352*	*<0.001*	*−9.030*	*0.019*	−4.408	0.397
	Land use	−2.986	0.311	−2.412	0.602	−4.325	0.395
*Enallagma cyathigerum*	Year	*−0.688*	*0.015*	*−1.065*	*0.001*	0.015	0.968
	Min. temp.	*−8.930*	*0.015*	−1.740	0.712	−6.612	0.080
	Land use	1.743	0.701	0.342	0.962	−1.966	0.687
*Erythromma najas*	Year	−0.352	0.184	*−1.007*	*0.041*	0.070	0.886
	Min. temp.	*−11.687*	*0.001*	−2.606	0.715	−4.730	0.246
	Land use	0.682	0.886	−1.410	0.886	−7.206	0.064
*Ischnura elegans*	Year	0.187	0.602	−0.409	0.272	0.109	0.604
	Min. temp.	*−8.698*	*0.013*	−2.026	0.701	−3.771	0.061
	Land use	−4.023	0.280	−3.689	0.498	−3.170	0.146
*Libellula quadrimaculata*	Year	−0.505	0.535	−1.032	0.204	−0.893	0.314
	Min. temp.	−13.072	0.064	−9.454	0.254	−11.227	0.225
	Land use	9.328	0.197	1.640	0.886	−2.524	0.857
*Orthetrum cancellatum*	Year	−0.198	0.625	*−0.863*	*0.040*	−0.580	0.064
	Min. temp.	*−9.018*	*0.040*	−1.389	0.881	−4.939	0.246
	Land use	5.228	0.246	2.878	0.687	−1.077	0.881
*Pyrrhosoma nymphula*	Year	−0.420	0.121	*−0.792*	*0.013*	0.026	0.962
	Min. temp.	*−12.678*	*<0.001*	*−9.474*	*0.014*	−6.552	0.157
	Land use	1.825	0.674	−1.777	0.701	−8.272	0.064
*Sympetrum striolatum*	Year	0.070	0.886	*0.521*	*0.008*	*0.973*	*0.014*
	Min. temp.	−1.926	0.692	1.511	0.573	2.456	0.674
	Land use	−0.321	0.962	−2.911	0.246	*−10.032*	*0.043*

Each coefficient value includes their corresponding *P* values adjusted using the FDR correction. Significant results are in italics

**Fig. 4:**
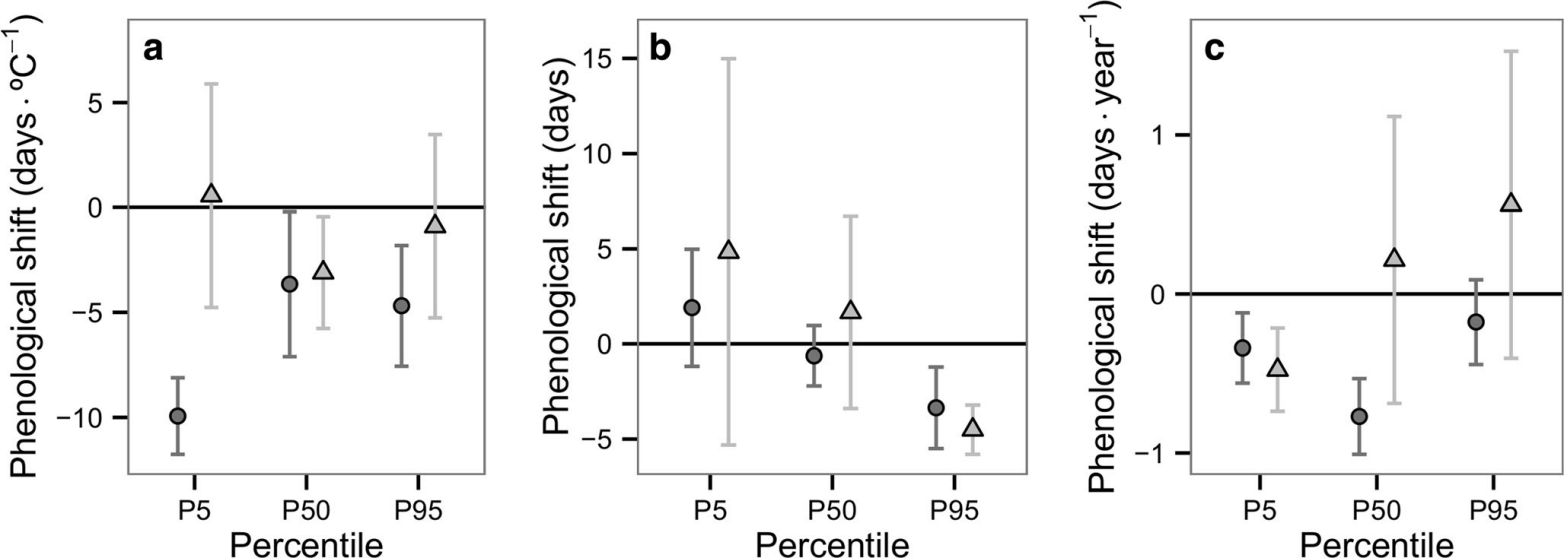
Shifts in flight dates (*P5*, *P50*, *P95*) in species with no egg diapause (*circle*), and obligate egg diapause (*triangle*). Shifts are in relation to **a** minimum temperature, **b** urban land use compared to the rural surroundings and **c** year. *Error bars* represent 95% confidence intervals

When the life history of odonates was included in the analysis, the presence or absence of a diapause was found to influence significantly the phenological response of odonates in relation to minimum temperature. Species without egg diapause showed a strongly significant advance in the P5 and P95 flight date compared to species with obligate egg diapause in response to minimum temperature (see Table 2; Fig. 4), as well as a small, but statistically significant shift in the P5 and P50 flight date according to year (Table 2). Species with and without egg diapause showed a significant advance in the P95 flight date in response to the UHI. However, all species with an obligate egg diapause tested belong only to the family Aeshnidae; therefore, it is difficult to ascertain the effects of an obligate egg diapause from the effects of phylogeny.

**Table 2:** Estimate of the mean phenological shift in days due to minimum temperature, urban land use and year according to life history traits

	No egg diapause					Obligate egg diapause				
	*N*	Mean	SE	*t*	*P*	*N*	Mean	SE	*t*	*P*
Min. temperature										
P5	9	−9.93	0.79	−12.530	*<0.001*	3	0.56	1.24	0.453	0.736
P50	9	−3.65	1.50	−2.442	0.097	3	−3.10	0.62	−5.010	0.097
P95	9	−4.68	1.25	−3.760	*0.025*	3	−0.89	1.02	−0.881	0.531
Urban land use										
P5	9	1.91	1.33	1.428	0.255	3	4.83	2.36	2.050	0.245
P50	9	−0.62	0.69	−0.904	0.487	3	1.66	1.17	1.416	0.376
P95	9	−3.35	0.93	−3.597	*0.027*	3	−4.51	0.30	−14.942	*0.023*
Year										
P5	9	−0.34	0.10	−3.552	*0.027*	3	−0.48	0.06	−7.844	0.052
P50	9	−0.77	0.10	−7.438	*0.001*	3	0.21	0.21	1.023	0.497
P95	9	−0.18	0.12	−1.533	0.236	3	0.56	0.22	2.502	0.212
	Spring species					Summer species				
	*N*	Mean	SE	*t*	*P*	*N*	Mean	SE	*t*	*P*
Min. temperature										
P5	6	−10.07	1.16	−8.658	*0.003*	6	−4.55	2.38	−1.910	0.206
P50	6	−3.34	2.04	−1.637	0.236	6	−3.68	1.13	−3.256	0.068
P95	6	−4.56	1.89	−2.414	0.121	6	−2.91	1.08	−2.694	0.097
Urban land use										
P5	6	3.74	1.28	2.925	0.091	6	1.54	1.98	0.778	0.531
P50	6	0.03	0.77	0.039	0.971	6	−0.13	1.10	−0.117	0.938
P95	6	−3.45	1.41	−2.449	0.121	6	−3.83	0.45	−8.512	*0.003*
Year										
P5	6	−0.42	0.06	−7.047	*0.005*	6	−0.32	0.14	−2.320	0.129
P50	6	−0.89	0.04	−19.761	*<0.001*	6	−0.16	0.23	−0.700	0.562
P95	6	−0.28	0.16	−1.744	0.222	6	0.29	0.16	1.862	0.209

Such traits considered are the presence/absence of egg diapause and spring/summer species. Each estimate of the phenological shift includes their corresponding *t* values and *P* values, which were adjusted using the FDR correction. Significant results are in italics

**Fig. 5:**
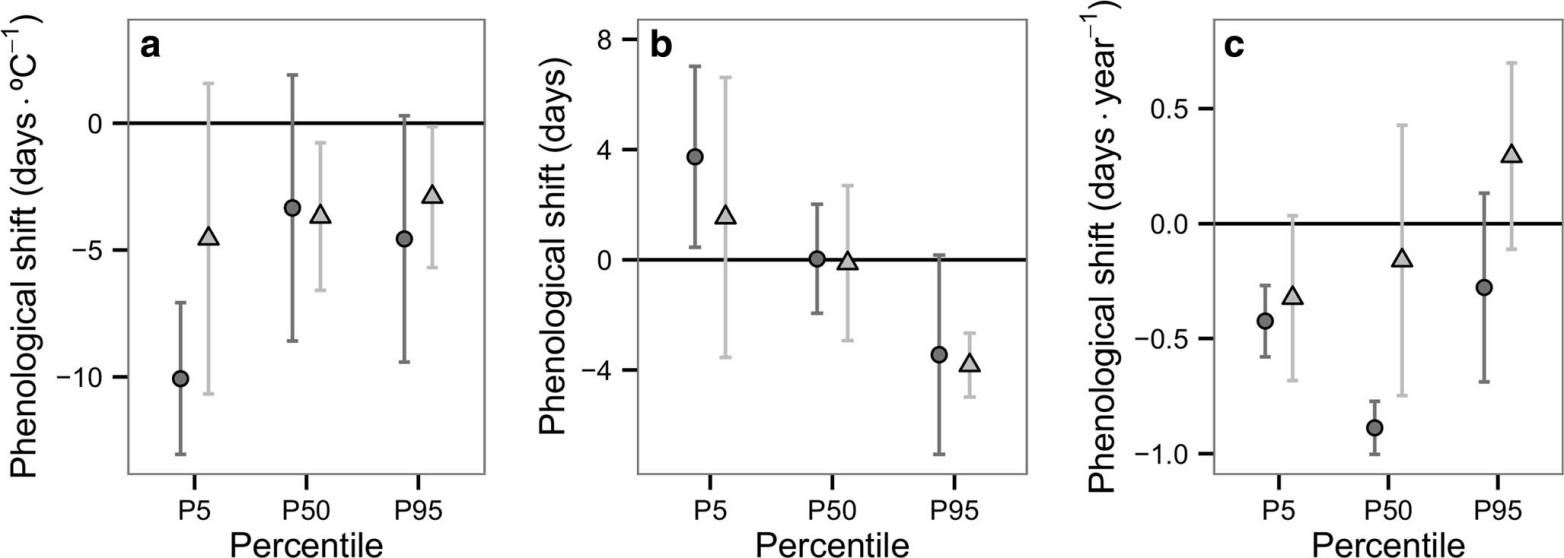
Shifts in flight dates (*P5*, *P50*, *P95*) in spring species (*circle*) and summer species (*triangle*). Shifts are in relation to **a** minimum temperature, **b** urban land use compared to the rural surroundings and **c** year. *Error bars* represent 95% confidence intervals

Regarding spring and summer species, spring species advanced their P5 flight date significantly in response to minimum temperature (Table 2; Fig. 5), as well as a significant advance in the P5 and P50 flight dates by year. On the other hand, summer species showed an advance in the P95 flight date in response to the UHI (Table 2; Fig. 5), thus suggesting a minor contraction of the flight period in the city within this group (Fig. 5). Summer species showed no significant shifts in their phenology in response to minimum temperature or year.

## Discussion

Our results suggest that, contrary to what might be predicted based on observations of the impacts of temperature on odonate phenology, the UHI only causes a slight advance in the trailing edge of the flight period at a community level with no change in the leading edge or middle flight date, and one species (*S. striolatum*) presented this advance in the P95 flight date. However, annual variation in minimum temperature appears to have a much stronger impact, with a clear advance of the flight period across the whole taxon and also in 50% of the species tested. Moreover, the life history of odonates imposes a great influence on the phenological response of the species to minimum temperature and, to a lesser extent, the UHI. In particular, both spring species and species without egg diapause tend to strongly advance their P5 flight date due to minimum temperature, whereas species with and without egg diapause advance the P95 flight date in response to the UHI to a minor extent. Additionally, summer species show an advance in their P95 flight date due to the UHI.

In previous studies, the UHI has demonstrated to have a strong impact on the phenology of terrestrial taxa (Cook et al. 2006; Deviche and Davies 2014; Jochner et al. 2013). However, spatio-temporal patterns in temperature, which was the quantitative measurement of the UHI used in this study, are driven by both the UHI and climate change. It is important to emphasise that the UHI implies a “local” temperature increase—although the extent of the UHI may depend on the city size and can be shifted by wind and topography (California Environmental Protection Agency 2015)—as opposed to climate change, the effects of which extend globally (IPCC 2013). The results from this investigation suggest that the UHI is only a minor contributor to thermal ecology of freshwaters, especially when compared to climate change. One explanation for this observation may be the presence of microclimates formed within and around urban freshwater ecosystems, which can buffer the effects of the UHI (Coutts et al. 2013; Hathway and Sharples 2012). Even though a study found the phenology of mosquitoes advanced in cities due to the UHI (Townroe and Callaghan 2014), this may be a result of the size of the water bodies investigated, which were only 80-l black plastic dustbins. The capability of water bodies to buffer the surrounding temperature depends greatly on the size of the water body (Jacobs et al. 2008). The external elements of urban water bodies may also contribute to buffer the impacts of the UHI. For example, a ring of trees around a pond provides a cooling effect (Forman 2014); therefore, ponds found within parks and/or woodlands will be less responsive to the UHI compared to other urban water bodies lacking surrounding vegetation. On the other hand, microclimates within urban water bodies are influenced by presence of aquatic vegetation and the impervious surface cover in the catchment (Paul and Meyer 2001). Despite urban water bodies often lacking riparian vegetation (Paul and Meyer 2001; Villalobos-Jimenez et al. 2016) to help regulate temperature, urban ponds in particular have a wide variety of features and are subject to different management plans, ranging from garden ponds to nature reserves (Hassall 2014), and contain a wide variety of invertebrate taxa as a result (Hill et al. 2016). Some urban ponds might be more effective at buffering the effects of the UHI, especially large urban ponds with abundant vegetation and decreased impervious cover in the catchment, and land managers can take advantage of this buffering capacity while preventing the establishment of invasive species and disease vectors (Hassall et al. 2016). The findings from this study may be a result of the *average* effectiveness of urban freshwater ecosystems at regulating the impacts of the UHI. However, the buffering capacity of water bodies would only be effective while the odonates develop in the aquatic environment; therefore, the adult phase—which is terrestrial—is more vulnerable to the impacts of the UHI, which may explain the advance of the P95 flight date in cities. However, the present study only considers one city (London) and its surroundings; therefore, further research is needed to analyse the biological implications of microclimatic conditions in urban water bodies in other cities.

Another factor to take into account is that urban climate is not only defined by increased temperatures—although it may be the most prominent feature—but it is also influenced by decreased insolation due to the buildings and other structures blocking the solar radiation (Terjung and Louie 1973). Likewise, the UHI is less intense in large areas dominated by green space (Forman 2014), where urban water bodies may have been found. Although the air temperature is still higher in cities despite having decreased insolation (Terjung and Louie 1973), this could potentially decrease the flight activity of odonates, which may in turn decrease detectability and recorder effort in urban areas.

Despite the fact that the community as a whole was advancing phenology in relation to changing temperature and showed a contraction of the flight period in urban areas, these patterns were not consistent across species. However, when the phenology was tested in the context of variation in life history traits, the results were more informative. For instance, species without egg diapause were found to be much more responsive to changes in minimum temperature compared to species with an egg diapause. The P5 flight date of species with no egg diapause advanced conspicuously in response to minimum temperature with a mean advance of 9.9 days °C^−1^. Likewise, the P95 flight date of species lacking an egg diapause showed a mean advance of 4.7 days °C^−1^, which suggests a shift of the flight period in response to minimum temperature. The lack of phenological shift in response to minimum temperature found in species with an egg diapause may be explained by the fact that the diapause protects the species from stressful conditions over winter but may also impede the species’ capacity to respond to increased warming in spring. Even though the sample size of species with an obligate egg diapause is rather small in this study—only three species, thus lacking statistical power—similar differences have been observed in the phenology of odonates in relation to climate change (Hassall et al. 2007) and even in other insect taxa such as aphids that overwinter in different stages (Harrington et al. 2007). On the other hand, species with and without an egg diapause responded similarly to the UHI by advancing the P95 flight date, suggesting that this trait does not help counteract the impacts of the UHI in the adult phase of odonates.

Similarly, spring species, which undergo a larval diapause in the last instar, show a considerable advance in the P5 flight date due to minimum temperature, which is in line with Hassall et al. (2007). However, the advance in the P95 flight date found in summer species in urban areas, as negligible as it may be, is somewhat unexpected since it is not in accordance with minimum temperature. The consequent temperature increase from the UHI is expected to extend the flight period instead of contracting the phenology. This contraction of the flight period of summer species in cities could be explained by increased mortality in the adult phase due to thermal stress, which is more noticeable in the summer compared to spring, or other stressors commonly found in urban areas, such as the presence of contaminants in water (Villalobos-Jimenez et al. 2016) that can also interact with the thermal stress even across metamorphosis (Janssens et al. 2014). However, further studies are required to decipher the mechanisms underlying this response.

## Conclusion

This investigation shows that the UHI does advance the final stage of the phenology of odonates, but only to a certain extent. However, the impacts of climate change on their phenology are far greater, with 50% of the species tested showing a clear advance in response to minimum temperature. The findings of this study provide evidence that climate change is the most important factor responsible for the increased temperatures and the subsequent phenological trends observed, whereas the UHI contributes only negligibly to the overall phenological trends. The present study also increases the current understanding of freshwater habitats and how their vulnerability towards climate change is probably higher than expected compared to the UHI. However, it is important to emphasise other factors in cities which can interact with increased temperatures and potentially alter the phenology of odonates and other aquatic insects, such as the presence of contaminants. Most importantly, the microclimatic conditions of urban water bodies can play an important role in regulating the effects of the UHI. Therefore, the biological impacts of the UHI can be dependent upon urban design and management (Hassall et al. 2016).

## Electronic supplementary material

ESM 1 (PDF 233 kb)
