# Supplementary material for: Effects of the urban heat island on the phenology of Odonata in London, UK
Source: Int J Biometeorol. 2017 Feb 11;61(7):1337–46. doi: 10.1007/s00484-017-1311-7 (PMC5486733; doi:10.1007/s00484-017-1311-7)
Supplement: Supplementary file 1 — (PDF 233 kb) [file 484_2017_1311_MOESM1_ESM.pdf]

Supplementary material:

Effects of the urban heat island on the phenology of Odonata in London, UK

International Journal of Biometeorology

Giovanna Villalobos-Jiménez†*, Christopher Hassall†

†School of Biology, University of Leeds, Woodhouse Lane, Leeds UK LS7 1SW

* Corresponding author: bsgdjv@leeds.ac.uk

Table S1. Variance Inflation Factors (VIF) of the predictor variables used in the FGLS models by species.

Species	Urban land use	Minimum temperature	Year
Aeshna cyanea	1.232	1.796	1.564
Aeshna grandis	1.095	1.124	1.029
Aeshna mixta	1.082	1.217	1.134
Anax imperator	1.171	1.283	1.112
Calopteryx splendens	1.064	1.112	1.048
Coenagrion puella	1.036	1.086	1.050
Enallagma cyathigerum	1.064	1.128	1.064
Erythromma najas	1.038	1.095	1.057
Ischnura elegans	1.097	1.228	1.131
Libellula quadrimaculata	1.057	1.108	1.051
Orthetrum cancellatum	1.092	1.094	1.002
Pyrrhosoma nymphula	1.068	1.160	1.092
Sympetrum striolatum	1.173	1.307	1.134

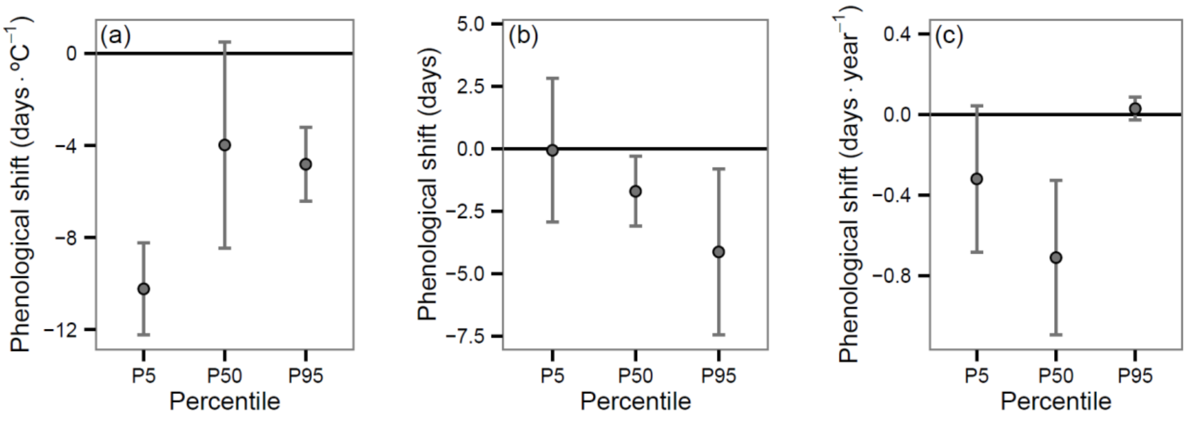

Figure S1. Community-level shifts excluding anisopterans in flight dates (P5, P50, P95) in relation to (a) minimum temperature, (b) urban land use compared to the rural surroundings, and (c) year. Error bars represent 95% confidence intervals.
